# *Staphylococcus aureus* Biofilm Growth on Cystic Fibrosis Airway Epithelial Cells Is Enhanced during Respiratory Syncytial Virus Coinfection

**DOI:** 10.1128/mSphere.00341-18

**Published:** 2018-08-15

**Authors:** Megan R. Kiedrowski, Jordan R. Gaston, Brian R. Kocak, Stefanie L. Coburn, Stella Lee, Joseph M. Pilewski, Michael M. Myerburg, Jennifer M. Bomberger

**Affiliations:** a Department of Microbiology and Molecular Genetics, University of Pittsburgh School of Medicine, Pittsburgh, Pennsylvania, USA; b Division of Pulmonary, Allergy and Critical Care Medicine, University of Pittsburgh School of Medicine, Pittsburgh, Pennsylvania, USA; c Department of Otolaryngology, University of Pittsburgh School of Medicine, Pittsburgh, Pennsylvania, USA; University of Kentucky

**Keywords:** biofilms, coinfection, host-pathogen interaction, polymicrobial

## Abstract

Staphylococcus aureus is a major cause of chronic respiratory infection in patients with cystic fibrosis (CF). We recently showed that Pseudomonas aeruginosa exhibits enhanced biofilm formation during respiratory syncytial virus (RSV) coinfection on human CF airway epithelial cells (AECs). The impact of respiratory viruses on other bacterial pathogens during polymicrobial infections in CF remains largely unknown. To investigate if S. aureus biofilm growth in the CF airways is impacted by virus coinfection, we evaluated S. aureus growth on CF AECs. Initial studies showed an increase in S. aureus growth over 24 h, and microscopy revealed biofilm-like clusters of bacteria on CF AECs. Biofilm growth was enhanced when CF AECs were coinfected with RSV, and this observation was confirmed with S. aureus CF clinical isolates. Apical conditioned medium from RSV-infected cells promoted S. aureus biofilms in the absence of the host epithelium, suggesting that a secreted factor produced during virus infection benefits S. aureus biofilms. Exogenous iron addition did not significantly alter biofilm formation, suggesting that it is not likely the secreted factor. We further characterized S. aureus-RSV coinfection in our model using dual host-pathogen RNA sequencing, allowing us to observe specific contributions of S. aureus and RSV to the host response during coinfection. Using the dual host-pathogen RNA sequencing approach, we observed increased availability of nutrients from the host and upregulation of S. aureus genes involved in growth, protein translation and export, and amino acid metabolism during RSV coinfection.

**IMPORTANCE** The airways of individuals with cystic fibrosis (CF) are commonly chronically infected, and Staphylococcus aureus is the dominant bacterial respiratory pathogen in CF children. CF patients also experience frequent respiratory virus infections, and it has been hypothesized that virus coinfection increases the severity of S. aureus lung infections in CF. We investigated the relationship between S. aureus and the CF airway epithelium and observed that coinfection with respiratory syncytial virus (RSV) enhances S. aureus biofilm growth. However, iron, which was previously found to be a significant factor influencing Pseudomonas aeruginosa biofilms during virus coinfection, plays a minor role in S. aureus coinfections. Transcriptomic analyses provided new insight into how bacterial and viral pathogens alter host defense and suggest potential pathways by which dampening of host responses to one pathogen may favor persistence of another in the CF airways, highlighting complex interactions occurring between bacteria, viruses, and the host during polymicrobial infections.

## Abstract (precis)

The airways of individuals with cystic fibrosis (CF) are commonly chronically infected, and Staphylococcus aureus is the dominant bacterial respiratory pathogen in CF children. CF patients also experience frequent respiratory virus infections, and it has been hypothesized that virus coinfection increases the severity of S. aureus lung infections in CF. We investigated the relationship between S. aureus and the CF airway epithelium and observed that coinfection with respiratory syncytial virus (RSV) enhances S. aureus biofilm growth. However, iron, which was previously found to be a significant factor influencing Pseudomonas aeruginosa biofilms during virus coinfection, plays a minor role in S. aureus coinfections. Transcriptomic analyses provided new insight into how bacterial and viral pathogens alter host defense and suggest potential pathways by which dampening of host responses to one pathogen may favor persistence of another in the CF airways, highlighting complex interactions occurring between bacteria, viruses, and the host during polymicrobial infections.

## INTRODUCTION

Staphylococcus aureus is the most frequently identified bacterial pathogen in cystic fibrosis (CF) respiratory infections. Due to lack of or to dysfunctional cystic fibrosis transmembrane regulator (CFTR) anion channels in epithelial cell membranes, numerous cell biological processes are dysregulated in individuals with CF, facilitating the establishment of chronic bacterial infections. Some factors that contribute to chronic CF respiratory infections include dehydrated airway surface liquid (ASL) and reduced mucociliary clearance, allowing for the buildup of thick mucus (1). Repeated infections elicit robust inflammatory responses dominated by elevated proinflammatory cytokines and continued accumulation of neutrophils; however, these inflammatory responses are ineffective at clearing pathogens in the CF lung, instead creating a hyperinflammatory cycle that leads to further tissue damage (2). In the past decade, S. aureus infection rates in CF patients have risen and have surpassed those of Pseudomonas aeruginosa, another prominent CF bacterial pathogen associated with worsening lung function (3). In 2015, over 70% of CF patients seen and cultured for respiratory pathogens in the United States were found to be infected with S. aureus, and methicillin-resistant S. aureus (MRSA) was identified in 26% of CF patients. Treatment options for S. aureus respiratory infections in CF include, but are not limited to, oral (linezolid, rifampin, clindamycin, and vancomycin) and intravenously (i.v.) administered (vancomycin and teicoplanin) antibiotics, and an inhaled vancomycin therapeutic option is currently undergoing clinical trials (4, 5). However, with the rising incidence of vancomycin-intermediate/resistant MRSA strains (VISA) and linezolid-resistant strains following antibiotic treatment, a better understanding of efficient ways to target and eradicate S. aureus chronic infections in CF is needed.

Along with chronic bacterial infections, CF patients also experience frequent respiratory virus infections. The most frequent viral pathogens identified in CF patient populations are respiratory syncytial virus (RSV), rhinovirus, influenza virus, parainfluenza virus, and adenovirus, with RSV and influenza virus infection linked to the greatest decreases in lung function (6–8). It was reported previously that close to 40% of young children with CF are hospitalized for severe respiratory infections, and respiratory viruses were identified in 50% of hospitalized patients, with RSV predominating (9). RSV infection may result in upper respiratory disease, including rhinitis, cough, fever, and acute otitis media, or progress to the lower respiratory tract, resulting in bronchiolitis or pneumonia in children, and exacerbate existing chronic airway disease in adults (10). RSV infection is especially aggressive in young infants with CF, leading to significant respiratory morbidity (11). Virus infection is believed to play a role in bacterial colonization in CF patients, with the majority of new bacterial colonization observed during peak virus season and in the weeks following upper respiratory tract infections (12). In pediatric CF populations, children experience respiratory virus infection at roughly the same frequency as adults, but their lower respiratory symptoms persist longer following acute virus infection (13). The respiratory microbiome in children with CF is dominated by S. aureus, and the median age of first infection with S. aureus is 3.6 years old (3). While little is known about the impact of virus coinfection on chronic S. aureus infections in CF, acute secondary S. aureus infection following respiratory virus infection in the non-CF population has been well studied. Influenza A virus infection can exacerbate existing S. aureus pneumonia (14) and promote transition of colonizing S. aureus from the nares to the lower respiratory tract, leading to development of pneumonia in mice (15). Infection with respiratory viruses stimulates the host innate immune response, leading to increased type I and III interferon (IFN) signaling. A recent study in mice showed that the type III interferon lambda (IFN-λ) response resulting from influenza virus infection led to increased susceptibility to S. aureus colonization and subsequent development of pneumonia (16). Given the impact that virus coinfection can have on S. aureus acute infections, we examined whether respiratory virus infections influence chronic biofilm infections with S. aureus.

Our recent work examined the effects of respiratory virus coinfection on P. aeruginosa biofilm growth in the CF airways. We observed that infection with RSV and other respiratory viruses enhanced P. aeruginosa biofilm growth on CF airway epithelial cells (AECs) (17). RSV infection induces an innate antiviral response in CF AECs mediated by IFN-λ, and peak P. aeruginosa biofilm biomass was observed when IFN-λ was most highly expressed. Addition of exogenous IFN-λ recapitulated the enhanced biofilm phenotype observed during virus coinfection. By evaluating CF AEC-secreted products, we observed that iron-bound transferrin was elevated during RSV infection, and removal of transferrin from CF AEC apical conditioned medium (CM) abrogated the biofilm-stimulatory effect in cell-free biofilm assays. We hypothesized that RSV coinfection may also impact interactions of S. aureus with the CF airway epithelium. To approach this question, we first adapted our polarized airway cell coculture model to evaluate growth of S. aureus strains in coculture with CF AECs. We observed increased S. aureus biofilm on RSV-infected immortalized CF AECs, as well as increased attachment and biofilm formation on primary CF human bronchial epithelial (HBE) cells, leading us to further investigate potential mechanisms mediating S. aureus-AEC interactions during virus coinfection.

## RESULTS

### S. aureus forms biofilms on polarized CF AECs.

**FIG 1:**
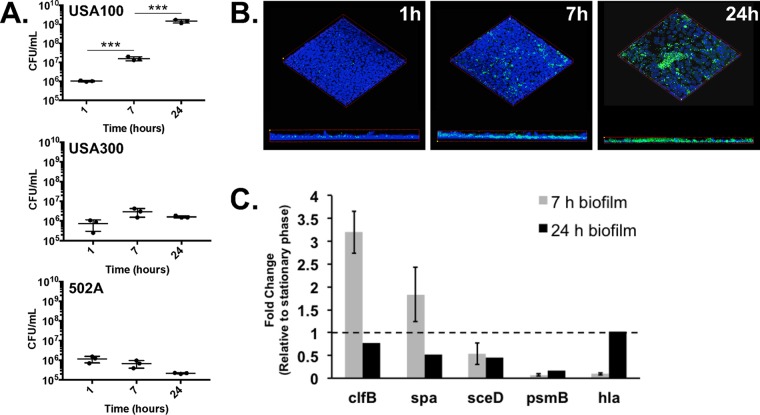
Characterizing S. aureus growth on cystic fibrosis airway epithelial cells. Coculture of S. aureus strains USA100, USA300, and 502A on polarized CF AECs over a 24-h time course (A). CFU per milliliter were measured at 1 h, 7 h, and 24 h postinfection. Polarized monolayers infected with GFP-expressing S. aureus USA100 (green) and then fixed and stained with Hoechst stain (blue) for confocal imaging at specified time points (B). Images show three-dimensional volume renderings at ×20 magnification generated in Nikon Elements. S. aureus gene expression on CF AECs alone and during RSV coinfection (C). Relative amount of mRNA transcript from S. aureus USA100 grown in coculture with control CF AECs or RSV-infected CF AECs, compared to S. aureus stationary-phase culture and measured by qRT-PCR. ***, *P* ≤ 0.001.

To assess the impact of RSV coinfection on S. aureus growth in the CF airways, we first adapted a model to evaluate S. aureus coculture with the CFBE41o- line of human ΔF508/ΔF508 cystic fibrosis bronchial airway epithelial cells (CF AECs) grown as a polarized monolayer. We apically infected CF AECs with three different S. aureus strains and observed growth over a period of 24 h (Fig. 1A). The hospital-associated methicillin-resistant S. aureus (MRSA) strain USA100, community-associated epidemic MRSA clone USA300, and human nasal colonizing isolate 502A all showed similar levels of attachment to CF AECs at 1 h postinoculation. The greatest increase in growth was exhibited by USA100 over 7-h and 24-h periods, while USA300 growth increased from 1 h to 7 h and then leveled off, and 502A growth decreased after initial attachment. Confocal scanning laser microscopy of green fluorescent protein (GFP)-expressing S. aureus showed biofilm-like clusters of USA100 forming on CF AECs by 7 h; however, by 24 h bacterial growth overwhelmed the CF AECs (Fig. 1B). Propidium iodide staining to indicate epithelial cell death and transepithelial electrical resistance (TEER) measurements of tight junction integrity indicated that the epithelial monolayer was intact after 1 h and 7 h of coculture with S. aureus but severely damaged by 24 h (see Fig. S1 in the supplemental material). Based on these observations, we pursued studies with USA100 and evaluated S. aureus biofilms after 7 h of coculture on CF AECs.

10.1128/mSphere.00341-18.1 FIG S1  Evaluating CF AEC epithelial barrier integrity during S. aureus coinfection. Transepithelial electrical resistance (TEER) measurements of S. aureus USA100 coculture with polarized CFBE41o- CF AECs in transwells over a 24-h time course (A). Measurements of uninfected control (Control, gray line) compared to S. aureus-infected CF AECs (USA100, red line). Propidium iodide staining of CFBE41o- cells cocultured with S. aureus (nonfluorescent) over a 24-h time course (B). Propidium iodide indicating dead/dying CF AECs, red; Hoechst stain of CF AEC nuclei, blue. Scale, 50 µm. Download FIG S1, TIF file, 2.8 MB. Copyright © 2018 Kiedrowski et al. 2018 Kiedrowski et al. This content is distributed under the terms of the Creative Commons Attribution 4.0 International license.

Several previous studies have characterized S. aureus gene expression during biofilm growth and colonization of the respiratory epithelium in various model systems, providing a reliable set of genes that may serve as biomarkers indicative of a biofilm state of growth (18, 19). To confirm that S. aureus exists in a biofilm state during growth on CF AECs in our model, we performed quantitative real-time PCR (qRT-PCR) on a panel of genes previously shown to be differentially expressed during planktonic and biofilm growth (Fig. 1C). We compared changes in expression of these genes in S. aureus cocultured on control and RSV-infected CF AECs. On control CF AECs, S. aureus showed significantly increased expression of surface adhesins (*clfB* and *spa*) after 7 h of growth, while expression of *agr*-regulated genes, including genes encoding factors for cell wall turnover (*sceD*) and secreted toxins (*psmB* and *hla*), decreased significantly compared to stationary-phase (planktonic) culture. By comparison, expression levels of *clfB*, *spa*, and *hla* were similar to stationary-phase levels after 24 h of growth on CF AECs. Taken together, these results confirm that S. aureus has transitioned to a biofilm-like state following 7 h of coculture with CF AECs.

### RSV infection promotes S. aureus biofilm growth.

**FIG 2:**
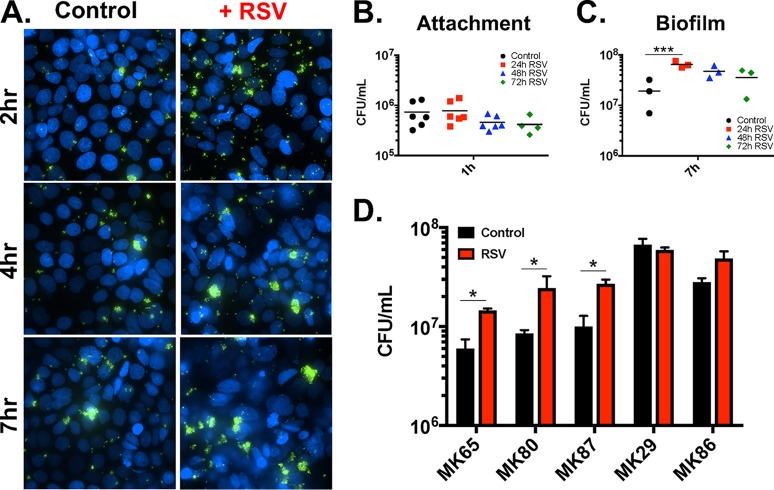
Effects of RSV coinfection on S. aureus attachment and biofilm growth on CF AECs. Live-cell imaging of S. aureus USA100 coculture with CF AECs in the absence and presence of 24-h RSV infection was observed using a closed flow-cell chamber and visualized on a Nikon Ti inverted wide-field microscope (A). Images were obtained at 2 h, 4 h, and 7 h after inoculation of S. aureus USA100 expressing GFP (green), with CF AEC nuclei stained with Hoechst stain (blue). Imaging experiments were repeated in triplicate, with 5 fields per chamber sampled. Static coculture biofilm assays with S. aureus USA100 were performed on polarized, air-liquid interface control CF AECs or CF AECs infected with RSV for 24 h, 48 h, or 72 h. Cocultures were allowed to proceed for 1 h to measure initial S. aureus attachment (B) and 7 h to measure biofilm growth (C). Bacteria were enumerated by plating after the desired incubation time and calculating CFU per milliliter. S. aureus clinical isolates cultured from sinonasal swabs collected from CF patients with chronic rhinosinusitis were evaluated in static biofilm coculture assays on CF AECs in the presence and absence of RSV infection (D). Black bars, control virus-free CF AECs; red bars, RSV-infected CF AECs. Experiments repeated in triplicate. Significance determined by unpaired Student’s *t* test; *, *P* ≤ 0.05; ***, *P* ≤ 0.001.

We previously observed that RSV coinfection promoted P. aeruginosa biofilms on CF AECs in a time-dependent manner, peaking at 72 h after infection with RSV. To determine if S. aureus growth was impacted by RSV, we infected immortalized CF AECs with RSV for 24, 48, or 72 h, followed by infection with S. aureus USA100 (Fig. 2). We observed similar levels of S. aureus attachment to uninfected and RSV-infected CF AECs at 1 h (Fig. 2A). Counts of CFU showed that S. aureus growth was increased on RSV-infected cells after 7 h of coculture, with the greatest increase in growth observed on CF AECs with 24-h RSV infections (Fig. 2B). Live-cell imaging of GFP-expressing S. aureus cocultured with CF AECs in a flow cell model showed enhanced formation of biofilm-like clusters of bacteria on RSV-infected CF AECs at 7 h of S. aureus growth compared to controls (Fig. 2C).

Hospital-associated MRSA clones like USA100 are still the most frequently isolated from CF patient populations (20); however, there is diversity in S. aureus clonal lineages carried by patients and isolated by clinical laboratories. To address this and assess if S. aureus strains broadly respond to RSV coinfection, we evaluated a group of S. aureus clinical isolates collected from adult CF patients with chronic rhinosinusitis (CRS) in coculture biofilm assays on CF AECs (Fig. 2D). Of five S. aureus CRS clinical isolates tested, four isolates (MK65, MK80, MK87, and MK86) showed significant increases in biofilm growth after 7 h of coculture on RSV-infected CF AECs. The isolate that did not show an increase in biofilm during RSV coinfection (MK29) already exhibited a log-greater biofilm growth under control conditions than other isolates evaluated, indicating that this isolate may possess unique factors for enhanced biofilm growth. These results are consistent with the conclusion that RSV coinfection promotes S. aureus biofilm growth at the mucosal surface of the CF airway epithelium.

### S. aureus attachment and biofilm growth are enhanced on primary CF HBE cells during RSV.

**FIG 3:**
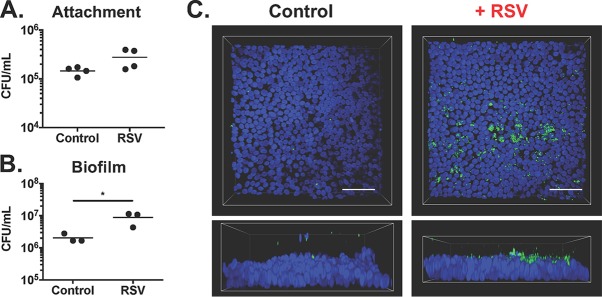
S. aureus attachment and biofilm growth on primary CF human bronchial epithelial cells during RSV coinfection. CFU counts of static biofilm coculture assays performed with S. aureus USA100 on primary CF HBE cells in the presence and absence of 72 h RSV coinfection at 1 h (A) and 7 h (B). Coculture assays were performed using at least two different CF HBE cell codes obtained from different patients and repeated for a minimum of *n* = 3 experiments. Significance determined by unpaired Student’s *t* test (*, *P* ≤ 0.05). GFP-expressing S. aureus USA100 (green) and CF HBE cell nuclei stained with Hoechst stain (blue) (C). Images were captured at ×60 magnification, with three-dimensional volume renderings and measurements obtained using Nikon Elements software. Scale, 20 µm.

Cell lines such as CFBE41o- CF AECs are a useful tool for studying CF disease; however, due to the process by which immortalized lines are developed from isolated cells, they may not entirely replicate the properties of the *in situ* airway epithelium. We next evaluated S. aureus biofilm formation in static coculture assays using primary, well-differentiated CF human bronchial epithelial (CF HBE) cells isolated from explanted lungs of CF patients at the University of Pittsburgh. Primary CF HBE cells were cocultured with S. aureus USA100 alone and during RSV coinfection, and attachment and biofilm formation were evaluated (Fig. 3). We observed an increase in S. aureus attachment to RSV-infected CF HBE cells after 1 h of coculture (Fig. 3A). Biofilm growth after 7 h of coculture on RSV-infected CF HBE cells was significantly increased compared to virus-free CF HBE cells (Fig. 3B). With confocal microscopy, we also observed an increase in S. aureus biofilm microcolony formation on primary CF HBE cells during RSV coinfection (Fig. 3C).

### Coinfection with hRV enhances S. aureus biofilm growth on CF AECs.

**FIG 4:**
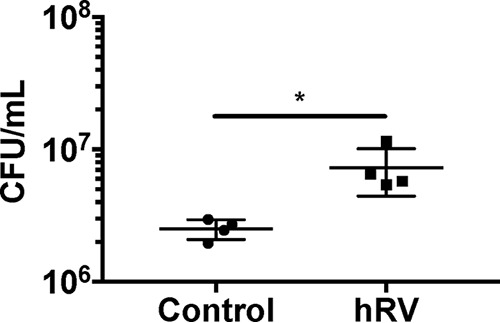
Coinfection with human rhinovirus enhances S. aureus growth on CF AECs. Static coculture biofilm assays with S. aureus USA100 were performed on polarized, air-liquid interface control CF AECs or CF AECs infected with human rhinovirus 14 (hRV) for 24 h at an MOI of 1. Cocultures were allowed to proceed for 7 h to measure biofilm growth. Bacteria were enumerated by plating and calculating CFU per milliliter. Significance was determined by unpaired Student’s *t* test (*, *P* ≤ 0.05).

We previously observed that enhancement of P. aeruginosa biofilms during virus coinfection was not specific to a particular respiratory virus, and biofilm growth was increased during coinfections with RSV, adenovirus, and human rhinovirus (hRV), all common CF respiratory pathogens (17). To determine if another respiratory virus could alter S. aureus biofilm growth in coculture, we performed biofilm assays using CF AECs infected with hRV 14 (Fig. 4). hRV coinfection significantly increased S. aureus growth on CF AECs, suggesting that the factors or mechanisms mediating this phenotype are not specific to a particular virus and, rather, are likely due to altered host processes during virus coinfection.

### S. aureus biofilms are enhanced in apical conditioned medium from RSV-infected CF AECs.

**FIG 5:**
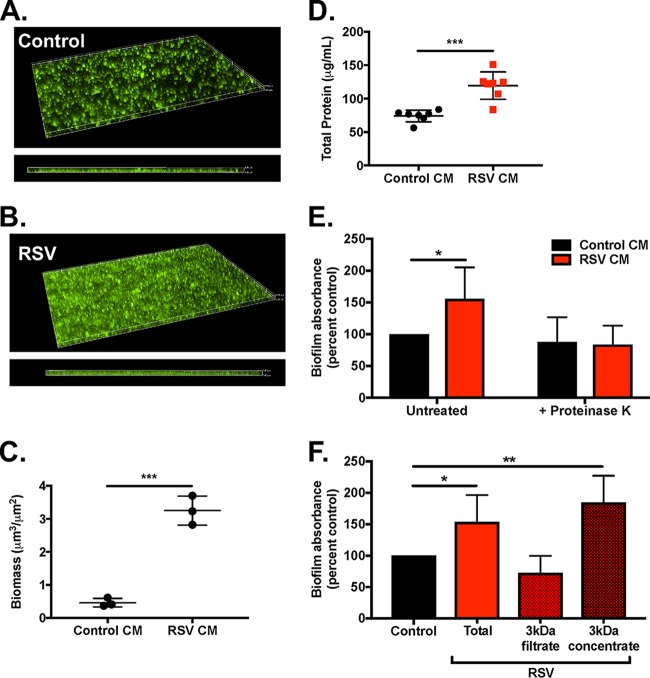
Conditioned medium from RSV-infected CF AECs enhances S. aureus biofilm growth. Conditioned medium containing apical secretions from polarized CF AECs was collected from control or RSV-infected cultures. GFP-expressing S. aureus USA100 was inoculated into CM and cultured in glass-bottom MatTek dishes for fluorescence microscopy. Microscopy was performed on a Nikon-Ti wide-field fluorescence microscope. Representative three-dimensional reconstructions of z-stacks obtained for S. aureus biofilm growth in control (A) and RSV (B) CM. Biomass quantification for control and RSV CM (C) was performed in Nikon Elements. Images and biomass quantification are representative of *n* = 3 individual experiments, with 5 random fields imaged per sample. Total protein content in CM and RSV CM (D). Biofilm growth in untreated control CM and RSV CM and CM pretreated with proteinase K (E) or subjected to 3-kDa centrifugal filtration (F). Biofilm assays were performed in microtiter plates, with a minimum of *n* = 3 biological replicates and 5 technical replicates per condition, per assay, with crystal violet absorbance quantified at 550 nm. *, *P* ≤ 0.05; **, *P* ≤ 0.01; ***, *P* ≤ 0.001.

RSV infection is known to stimulate the innate immune response in the airway epithelium, a process mediated by interferon (IFN) signaling. As IFNs are secreted factors, we previously tested P. aeruginosa biofilm growth in conditioned medium (CM) collected from the apical surface of polarized CF AECs to learn if secreted factors influenced biofilm growth. We found that CM from RSV-infected CF AECs stimulated P. aeruginosa biofilms, compared to control CM from virus-free CF AECs (17). To examine if S. aureus-enhanced biofilm growth during virus coinfection may be due in part to secreted factors, we collected CMs from control and RSV-infected polarized CF AECs, inoculated the CM samples with GFP-expressing S. aureus USA100 in polylysine-coated glass-bottom MatTek dishes, and used fluorescence microscopy to measure biofilm biomass (Fig. 5). We observed significantly enhanced S. aureus biofilm growth in RSV CM compared to control CM from virus-free CF AECs, with S. aureus forming a thick, confluent biofilm in RSV CM (Fig. 5A and B). Biomass quantification also showed a significant difference between biomass measured in RSV and that in control CM (Fig. 5C). This suggests that factors secreted from CF AECs during virus coinfection can stimulate S. aureus biofilm growth in the absence of direct contact with the airway epithelium.

We performed an initial characterization of RSV CM to better understand the nature of biofilm-stimulatory secreted factors present. We found that the total protein content of RSV CM is significantly increased (*P* < 0.001) compared to control CM (Fig. 5D). Hypothesizing that an increase in total protein content or release of specific proteins during virus infection may facilitate S. aureus-enhanced biofilm growth, we pretreated CM with proteinase K and evaluated biofilm formation in microtiter plate assays (Fig. 5E). The significant increase in biofilm usually observed in RSV CM was lost in proteinase K-treated RSV CM, whereas biofilm growth in control proteinase K-treated CM was not affected. To gain an estimate of the size of the protein(s) involved in biofilm stimulation, control and RSV CMs were centrifuged using 3-kDa-cutoff filters, and biofilm assays were performed using the contents of the flowthrough (filtrate) and material retained by the filter (concentrate) (Fig. 5F). Biofilm growth in control CM was not altered in either the 3-kDa filtrate or 3-kDa concentrate compared to total CM. However, growth in 3-kDa filtrate from RSV CM reduced biofilm biomass down to levels observed in control CM, whereas 3-kDa concentrate from RSV CM supported significantly enhanced biofilm growth. These results suggest that a protein or proteins greater than 3 kDa secreted from CF AECs during virus infection may serve to enhance S. aureus biofilm growth at the airway epithelial surface.

### Addition of iron does not impact S. aureus biofilms on CF AECs.

**FIG 6:**
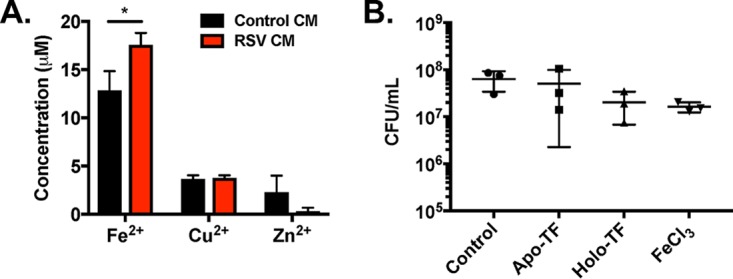
Addition of iron does not enhance S. aureus biofilms on CF AECs in the absence of virus infection. Total concentration of metals measured in CM and RSV CM, as measured by individual assay kits for iron (Fe^2+^), zinc (Zn^2+^), and copper (Cu^2+^) (A). CFU counts from static biofilm coculture assays performed with S. aureus USA100 on CF AECs in MEM plus 2 mM l-glutamine alone or with addition of exogenous 250 µM FeCl_3_, 12.5 µM apotransferrin (non-iron bound), or 12.5 µM holotransferrin (iron bound) diluted in MEM plus 2 mM l-glutamine (B).

We previously showed that apical levels of iron-bound transferrin are increased during RSV infection, and transferrin was found to be a key factor responsible for enhancing P. aeruginosa biofilms in our CF AEC model. Like P. aeruginosa, S. aureus also encodes several factors for host iron acquisition, and when we observed that RSV CM increased S. aureus biofilms, we hypothesized that iron may again be the secreted factor affecting biofilm formation. First, to determine if homeostasis of other metals is disrupted by RSV infection, we measured concentrations of iron, zinc, and copper in control and RSV CMs (Fig. 6A). Only iron levels were found to be significantly increased during RSV infection in CF AECs. Next, to assess if increased iron availability during coculture with CF AECs impacts S. aureus biofilms, we performed biofilm assays on CF AECs in the absence of virus infection and added exogenous free iron (FeCl_3_), non-iron-bound transferrin (apotransferrin [apo-TF]), and iron-bound transferrin (holotransferrin [holo-TF]) (Fig. 6B). We observed no significant changes in S. aureus biofilms on CF AECs with addition of FeCl_3_, holotransferrin, or apotransferrin. These data suggest that increases in apical iron observed during RSV infection that potentiate P. aeruginosa biofilm growth (17) are not a factor in promoting S. aureus biofilms during virus coinfection.

### Dual host-pathogen RNA-seq reveals changes in S. aureus and CF AEC gene expression during RSV coinfection.

**FIG 7:**
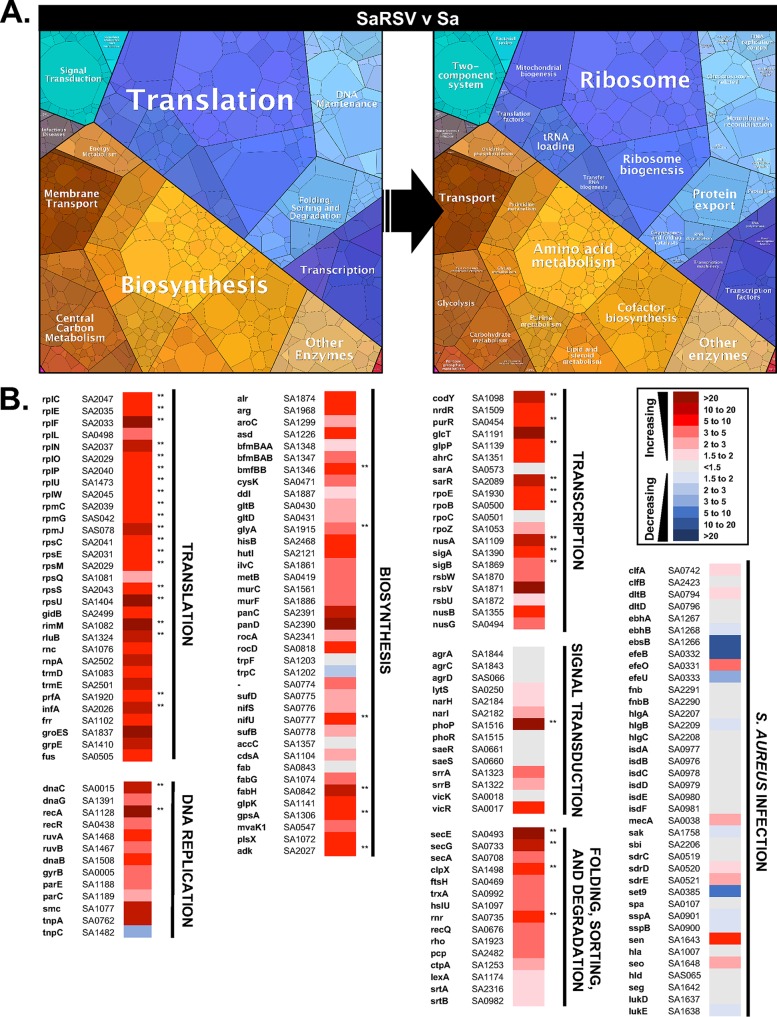
S. aureus transcriptomic changes during coculture on RSV-infected CF AECs. Proteomaps depicting functional categories of differentially expressed S. aureus genes during S. aureus-RSV coinfection compared to S. aureus single infection of CF AECs (SaRSV v Sa) (A). Heat maps showing fold change gene expression in specific S. aureus genes during S. aureus-RSV coinfection compared to S. aureus single infection of CF AECs (B). In each heat map, column 1 shows gene name, column 2 shows S. aureus gene identifier (from S. aureus strain N315 genome annotation), column 3 shows fold change relative to single infection, and column 4 indicates false discovery rate *P* value significance, with ** indicating a false discovery rate *P* value of < 0.05.

**FIG 8:**
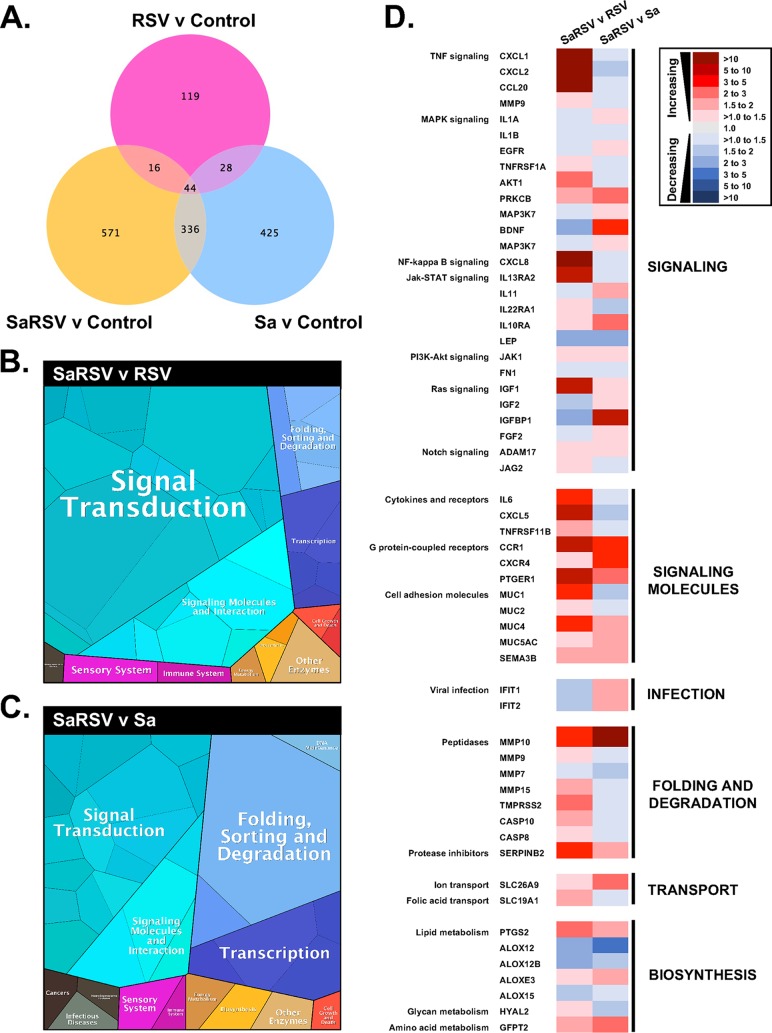
Transcriptomic changes in CF AECs during coinfection with S. aureus and RSV. Venn diagram depicts differentially expressed transcripts under each of RSV (RSV v Control, pink), S. aureus (Sa v Control, blue), or S. aureus-RSV coinfection (SaRSV v Control, yellow) conditions (A). Proteomaps depict functional categories of differentially expressed host genes during S. aureus-RSV coinfection compared to either RSV (SaRSV v RSV) (B) or S. aureus (SaRSV v Sa) (C) single infections. Heat maps indicate fold change gene expression under SaRSV v RSV or SaRSV v Sa conditions (D).

Our observations of increased S. aureus biofilm growth on CF AECs and in CF AEC apical secretions (CMs) suggest that processes in S. aureus, the airway epithelium, or both are altered during respiratory virus coinfection. To gain insight into key pathways that are differentially regulated during a bacterial-viral polymicrobial infection, from the perspective of both S. aureus and the CF airway epithelium, we performed dual host-pathogen RNA sequencing (RNA-seq) on samples collected from coculture biofilm assays (Fig. 7 and 8). RNA was collected from uninfected (control), RSV-infected, S. aureus-infected, or RSV-S. *aureus*-coinfected CF AECs, and sequencing reads were mapped to both the S. aureus and human genomes. Analyses were then performed to compare differential gene expression in S. aureus during coculture with virus-free or RSV-infected CF AECs and in CF AECs during infection with S. aureus compared to coinfection with both S. aureus and RSV.

In S. aureus, 1,308 genes were found to be differentially expressed (1.5-fold or greater) during growth on CF AECs coinfected with RSV, compared to growth on CF AECs in the absence of virus. Of these, 123 genes had significantly different expression (*q* ≤ 0.05, as determined by Rockhopper analysis). Pathway analysis showed that many of the most highly upregulated S. aureus genes during RSV coinfection were related to translation and biosynthesis (Fig. 7A). Associated with protein translation, genes for ribosomal processes and ribosome biogenesis were significantly upregulated. Within biosynthesis pathways, genes for amino acid metabolism and lipid metabolism were found to be highly upregulated. Significantly upregulated genes in other S. aureus functional pathways included those related to homologous recombination (*recA*), protein export (*secE*) and protein folding (*groES*), two-component signal transduction (*phoP*), membrane transport (*oppF*, *rlp*, and *mnhC*), and carbohydrate metabolism (*glmU*) (Fig. 7B). Several global transcriptional regulators also showed increased expression during RSV coinfection, including those related to transcription machinery (*sigB* and *sigA*), RNA polymerase (*rpoB* and *rpoE*), and transcription factors (*codY*, *purR*, *glcT*, and *ahrC*). The pattern of upregulated genes suggests that S. aureus responds to RSV coinfection by growing and producing proteins, and yet the findings of increased transcript for the transcriptional regulator *sarR* and the two-component system *srrAB*, known regulators of the *agr* quorum sensing system, and no observed increase in transcript for *agr* genes suggest that despite indicators of growth, *agr* quorum sensing remains repressed, keeping S. aureus in a biofilm-like state.

Analysis of CF AEC transcriptomes during infection showed that compared to control (uninfected) CFBE41o- cells, RSV infection led to 207 differentially expressed transcripts (119 unique), S. aureus infection led to 833 differentially expressed transcripts (425 unique), and S. aureus-RSV coinfection led to 967 differentially expressed transcripts (571 unique) (Fig. 8A). Analysis of coinfection transcriptomes compared to either RSV or S. aureus single infections using Ingenuity Pathway Analysis allowed for identification of pathways of interest altered by each pathogen during coinfection, and the Proteomaps treemaps algorithm (21) allowed visualization of proteins related to specific cellular function (Fig. 8B). Comparing fold change gene expression in S. aureus-RSV coinfection to either RSV or S. aureus single infections frequently showed divergent responses to infection (Fig. 8D). S. aureus was found to upregulate genes related to tumor necrosis factor (TNF) signaling (CXCL1, CXCL2, and CCL20) and other immune signaling pathways (CXCL8 and IL13RA2), cytokine and receptor interactions (interleukin-6 [IL-6] and CXCL5), G-protein-coupled receptors (CCR1 and PTGER1), several peptidases (MMP10, MMP9, MMP15, TMPRSS2, CASP10, and CASP8), and some airway mucins (MUC1 and MUC4) when comparing coinfection to RSV infection alone. However, a comparison of RSV infection alone and coinfection with S. aureus found downregulation of the same genes involved in cell signaling and cytokine-receptor interactions, as well as all peptidases, except MMP10. RSV induced increased expression of antiviral genes (IFIT1 and IFIT2), as well as some genes involved in mitogen-activated protein kinase (MAPK) signaling (PRKCB and BDNF) and G-protein-coupled chemokine receptor signaling (CCR1 and CXCR4). Taken together, we see that S. aureus and RSV make different, and often divergent, contributions to the host response to polymicrobial infection.

## DISCUSSION

We previously observed that P. aeruginosa, a leading CF pathogen, responded to respiratory virus coinfection by enhancing biofilm formation on CF AECs (17). In this work, we show that another major bacterial pathogen in CF, S. aureus, also exhibits increased biofilm growth on CF AECs during virus coinfection. Like P. aeruginosa, S. aureus biofilms were also enhanced in conditioned medium from RSV-infected cells, but while iron was found to be a major host-secreted factor impacting P. aeruginosa biofilms (17), S. aureus did not respond significantly to iron addition, suggesting that different aspects of the host response to virus infection impacted S. aureus biofilm growth. Analysis of factors in RSV CM responsible for S. aureus biofilm stimulation suggests that the unknown factors are not small molecules, peptides, or free amino acids. To gain a better understanding of the processes underlying S. aureus interactions with CF AECs during RSV coinfection, we harnessed the power of dual host-pathogen RNA sequencing to provide a broad view of changes occurring in both S. aureus and the CF airway epithelium. This method allowed us to observe specific contributions of each pathogen and brought to light potential mechanisms altered in both the host and S. aureus in the presence of virus coinfection.

During our initial adaptation of a static biofilm coculture model on polarized CF AECs, we evaluated methicillin-resistant S. aureus strains representative of clonal lineages associated with both hospital- and community-acquired human infections, as well as the nasal colonization isolate 502A. Previous work with USA300, an epidemic community-associated MRSA clone, showed that high levels of alpha-toxin produced during coculture with polarized Calu-3 airway epithelial cells at the air-liquid interface led to early loss of barrier integrity and killing of the AECs in less than 24 h (22). In this work, we found that a hospital-associated MRSA strain, USA100, formed robust biofilms on CF AECs after 7 h of coculture without inducing significant damage to the epithelial monolayer. USA100 is a strain lineage associated with S. aureus clonal complex 5, a group commonly found colonizing the nasal passages of people in the United States and whose members are well known for causing invasive disease and chronic infections, including endocarditis and persistent respiratory infections in CF (20, 23). Quantitative PCR (qPCR) confirmed that USA100 expresses low levels of alpha-toxin at 7 h in coculture with CF AECs, and imaging studies showed formation of biofilm-like clusters of S. aureus USA100 present on the apical surface of CF AECs. This trend reverses after 24 h of coculture, with alpha-toxin expression increasing. This timing agrees with our previous observation that by 6 h of coculture in our model, P. aeruginosa forms similar microcolony structures on CF AECs (17, 24). While both P. aeruginosa and S. aureus biofilms formed on CF AECs do not resemble thicker, confluent biofilm structures that can be achieved in non-host-associated biofilm models, gene expression studies confirmed that both bacteria do exist in a biofilm state on CF AECs during this stage of growth in coculture.

It has long been hypothesized that RSV and other respiratory viruses play a role in promoting S. aureus pathogenesis in CF lung infections (25), but specific mechanisms mediating S. aureus-virus interactions in CF have yet to be revealed. We observed that coinfection with RSV or rhinovirus, two common viral pathogens in CF, resulted in increased S. aureus biofilm growth on a CF lung epithelial cell line and on primary well-differentiated CF human bronchial epithelial cells. Respiratory virus infections affect the airway epithelium in many ways, including by inducing cell death (26), stimulating the innate antiviral response (27, 28), and leading to dysregulated nutrient secretion (17), which can potentially impact coinfecting bacteria. We observed that apical conditioned medium (CM) from polarized RSV-infected CF AECs promoted S. aureus biofilm growth in the absence of direct contact with epithelial cells, suggesting that S. aureus benefits from a secreted factor produced by virus-infected AECs. We previously found that increased levels of apical secreted iron, in the form of host iron-bound transferrin, during virus coinfection benefit P. aeruginosa biofilms (17). S. aureus produces two Fur-regulated siderophores, staphyloferrins A and B (29), that are capable of stealing iron from host transferrin *in vitro* (30). Unlike P. aeruginosa, some S. aureus strains have been found to form more robust biofilms under iron-depleted conditions *in vitro* (31), and excess iron instead leads to activation of several virulence genes and promotes an antibiofilm state (32). We observed that in coculture with CF AECs exogenous addition of several different iron sources at physiologically relevant concentrations did not significantly alter S. aureus USA100 biofilms. This result suggests that iron may not be a significant factor impacting S. aureus during CF lung infections. We see that biofilm formation by S. aureus CF clinical isolates ranges during virus coinfection, and the possibility remains that diverse S. aureus strain lineages may respond differently to virus-induced available iron or other nutrients during coinfection in the CF lung, potentially affecting biofilm formation or interactions with the host epithelium or other resident respiratory microbes.

The application of next-generation sequencing and omics technologies to CF studies has already highlighted the complex microbial environment that exists in the CF lung (33, 34), and advances in RNA sequencing and analysis now allow an unprecedented opportunity to gain insight on changes in both host and pathogen gene expression during infection. Here, we used a dual host-pathogen RNA-seq approach to evaluate transcriptomic changes in CF AECs and S. aureus during coinfection with RSV. Performing individual S. aureus and RSV infections and comparing these conditions to an S. aureus-RSV coinfection lets us begin to decipher what host responses may be due to each respective pathogen in our model. Comparing the transcriptome of S. aureus cocultured with control to that of *S. aureus* cocultured with RSV-infected CF AECs, we found increased levels of transcripts for genes related to translation and biosynthesis, indicating that S. aureus is actively producing proteins and metabolizing in association with a virus-infected host substratum. Expression of the *agr* quorum sensing system genes does not change during RSV coinfection, and increased levels of the transcriptional regulator SarR suggest that SarR-mediated repression may be involved in keeping S. aureus in a biofilm-like state (35, 36). Further repression of *agr* may be mediated by the SrrAB two-component system (37), also upregulated in RSV coinfections. Interestingly, SrrAB is known to be active under decreased oxygen conditions (37), and as the CF lung is known to become increasingly anoxic as airways become occluded by chronic bacterial biofilm infections and mucus over time, this system may have an important role in S. aureus during coinfections. Supporting our observations that iron addition did not significantly impact S. aureus biofilms on CF AECs, transcriptomic analysis shows that S. aureus iron acquisition systems (*efe* system [38] and *isd* genes) are not upregulated during RSV coinfection. We observed that CM from RSV-infected AECs stimulated S. aureus biofilms, suggesting that a host-secreted factor may affect biofilm growth. Our studies characterizing biofilm-stimulatory factors in RSV CM suggested that increased high-molecular-weight proteins secreted during virus infection may be utilized by S. aureus to enhance biofilm growth. Correspondingly, we see that several S. aureus genes for catabolism of amino acids significantly increase during RSV coinfection, suggesting that a host secreted protein may be recognized and utilized by S. aureus, and future studies will investigate if virus infection leads to increased secretion of specific proteins into the ASL or to altered availability of other nutrients at the mucosal interface.

Using dual RNA-seq to evaluate changes in the host CF airway epithelial transcriptome, we first observe by comparing S. aureus and RSV single infections and S. aureus-RSV coinfection to control CF AECs that each infection condition induces a largely unique response in the epithelium (Fig. 8A). Each condition has more unique differentially regulated transcripts than transcripts that overlap another infection condition, as may be expected when comparing responses to diverse bacterial and viral pathogens. We next compared S. aureus-RSV coinfections directly to each single-infection condition, to gain an understanding of what the specific contribution of each pathogen may be to the overall host response in a coinfection setting. We see broadly that induction of different functional groups of genes may be attributed to S. aureus (“SaRSV v RSV” analysis in figures) and RSV (“SaRSV v Sa” analysis). S. aureus has a proinflammatory signature, with increased TNF and cytokine signaling during coinfection (Fig. 8D). This response is conversely downregulated by RSV during coinfection. RSV was found to promote an increase in interleukin-10 receptor A during coinfection. While IL-10 is not present in our model, it was recently observed that in localized infections, IL-10 promotes S. aureus persistence (39), suggesting that RSV coinfection in the CF lung where immune cells are abundant could impact chronic S. aureus infection. Interestingly, it appears that S. aureus decreases expression of the interferon-stimulated antiviral genes IFIT1 and IFIT2 during RSV coinfection, compared to RSV infection alone, where both IFIT1 and IFIT2 are increased, as predicted. Mucins are known to mediate numerous bacterial interactions with epithelial surfaces (40, 41). Our data agree with previous studies showing that RSV (42, 43) and S. aureus (42) increase MUC5AC expression, and we also observe that S. aureus infection increases expression of the periciliary-layer mucins MUC1 and MUC4 (Fig. 8D). Taken together with our data that host-secreted products of >3 kDa produced during RSV infection stimulate S. aureus biofilm growth, changes in airway mucin production during coinfection and their impact on S. aureus warrant further study. Collectively, the dual RNA-seq results suggest that S. aureus can dampen the host response to viral pathogens, which could have important implications for management of CF respiratory infections, and altered expression of airway mucins could impact S. aureus interactions with the airway epithelium.

Together, this work has characterized the interactions of S. aureus and RSV coinfection in our polarized CF AEC coculture model. Transcriptomic analysis allows us to observe that S. aureus and RSV each have specific contributions to the host epithelial response during coinfection. Differential expression of innate immune genes shows that S. aureus can alter the host response to a respiratory virus, and vice versa, changing how the host defends against diverse pathogens during coinfection. What we have learned from this work examining S. aureus-RSV coinfections of CF AECs sets the stage for future studies that will allow us to better understand complex bacterium-bacterium and bacterium-host interactions taking place in the CF lung and how these relationships are impacted by coinfection with respiratory viruses.

## MATERIALS AND METHODS

### Bacterial and virus strains, cell lines, and growth conditions.

S. aureus strains were cultured in tryptic soy broth (TSB) rotating overnight at 37°C, with 10 µg/ml chloramphenicol when needed for plasmid maintenance. S. aureus strains used include the USA100 Tokyo clone (43), USA300 LAC (44), and 502A (45). For microscopy studies, USA100 carrying the GFP-expressing vector pCM29 was used (46). CF chronic rhinosinusitis clinical isolates were obtained from sinonasal swabs collected during endoscopic sinus procedures that were clinically indicated for management of patients’ chronic sinusitis (University of Pittsburgh IRB REN16110185). Swabs were streaked to tryptic soy agar (TSA) with 5% sheep’s blood containing colistin and nalidixic acid and cultured for 24 to 48 h at 37°C. Single colonies were used to inoculate overnight cultures, and genomic DNA was prepared from cultures using a DNeasy Blood and Tissue kit (Qiagen) with lysostaphin pretreatment for 1 h at 37°C. PCR was performed on genomic DNA using universal bacterial 16S primers 63F (5′ CAGGCCTAACACATGCAAGTC 3′) and 1387R (5′ GGGCGGAGTGTACAAGGC 3′) (47), and resulting PCR products were sequenced by Eurofins Genomics. NCBI DNA BLAST searches confirmed the species matched to S. aureus.

Coculture biofilm assays were performed with the immortalized human ΔF508/ΔF508 CF bronchial epithelial cell line CFBE41o- (CF AECs). CF AECs were cultured on transwell filters and grown at the air-liquid interface for 7 to 10 days. Primary CF HBE cells were obtained from explanted lungs of CF patients with informed consent under a protocol approved by the Institutional Review Board at the University of Pittsburgh. Primary CF HBE cells were cultured on transwell inserts under air-liquid interface conditions.

For virus infections, the purified human A2 strain of RSV was used at a multiplicity of infection (MOI) of 1. For infections with human rhinovirus, the purified human rhinovirus 14 strain was used at an MOI of 1 for 24 h.

### Static coculture biofilm assays.

Static coculture biofilm assays with S. aureus and CF AECs or primary CF HBE cells were performed as previously described for P. aeruginosa with some modifications (17). Control AECs or AECs infected with RSV for 24 h, 48 h, or 72 h were inoculated with S. aureus at an MOI of ~250. Apical medium was removed, unattached bacteria were washed off after a 1-h incubation, and biofilms were grown for an additional 6 h or 23 h at 37°C in 5% CO_2_. For iron addition coculture assays, bacteria were inoculated onto CF AECs in the presence of either minimal essential medium (MEM) alone or MEM with 250 µM FeCl_3_, 12.5 µM apotransferrin, or 12.5 µM holotransferrin. Filters were washed once, and CFU were counted by lysing epithelial cells, dissociating bacterial biofilms with 0.1% Triton X-100, and dilution plating to tryptic soy agar plates.

### Confocal imaging of static coculture biofilms.

To image coculture biofilms on transwell filters, biofilm assays were performed as described above with S. aureus USA100 expressing GFP. At the conclusion of the assay, CF AECs on transwell filters were washed once, stained with Hoechst stain (1:1,000 in MEM) for 10 min, and then fixed in cold 4% paraformaldehyde (PFA; diluted in phosphate-buffered saline [PBS]) overnight at 4°C. For live/dead staining of CF AECs during S. aureus and virus coinfections, filters were stained with propidium iodide solution (Sigma-Aldrich) diluted 1:8,000 in MEM for 15 min prior to washing and fixing. After fixing, PFA was removed and filters were washed once with cold PBS. Transwell filters were cut out from inserts, mounted on slides in ProLong Gold antifade reagent, sealed with coverslips, allowed to cure at room temperature for 24 h in the dark, and then stored at 4°C. Microscopy of fixed filters was performed on an Olympus FluoView FV1000 inverted confocal microscope. Bacterial biomass was quantified with Nikon Elements software.

### qRT-PCR of S. aureus genes.

To prepare RNA for quantitative real-time PCR (qRT-PCR), CF AECs and S. aureus were harvested in PBS after 7-h and 24-h static biofilm coculture and pelleted by centrifugation. PBS was removed, and pellets were resuspended in RNAlater and incubated overnight at 4°C. Bacteria were then pelleted by centrifugation and resuspended in cold RNA-Bee reagent (Amsbio) on ice. Samples were transferred to cold screw-cap tubes with 0.1-mm zirconia-silica beads, mechanically lysed in a Mini-Beadbeater-24 (BioSpec Products) at maximum speed for 1 min, and then incubated on ice for 1 min, with bead beating and cooling steps repeated three times. An 0.2-ml volume of chloroform was added to each sample and shaken vigorously, and then samples were centrifuged at 12,000 relative centrifugal force (RCF) for 15 min at 4°C. The aqueous phase was transferred to a clean tube with 0.5 ml isopropanol and inverted to mix, and 2 µl linear acrylamide was added. Samples were centrifuged at 12,000 RCF for 5 min at 4°C, and supernatants were removed. Resulting RNA pellets were washed with 75% ethanol, allowed to dry, and resuspended in sterile RNase-free water. RNA concentrations were measured on a NanoDrop 2000 UV-Vis spectrophotometer. As a control, S. aureus USA100 cultures were grown overnight in tryptic soy broth (TSB) to stationary phase and then normalized to an optical density at 600 nm (OD_600_) of 1.0, and RNA from 1 ml of normalized culture was extracted with RNA-Bee as outlined above. For each sample, cDNA was generated from 1,000 ng RNA with iScript reverse transcriptase (Bio-Rad) and then diluted 1:3. Quantitative real-time PCRs were performed using iQ SYBR Green Supermix (Bio-Rad) in a Bio-Rad CFX Connect real-time PCR detection system. Oligonucleotides were synthesized by Sigma using previously published sequences to S. aureus planktonic and biofilm markers (22). Transcript levels were calculated using quantification cycle (ΔΔ*C*_*q*_) with DNA gyrase reference gene (*gyrB*).

### Live imaging of flow-cell coculture biofilms.

Live-imaging experiments were performed based on methods previously described for P. aeruginosa biotic biofilms (17). Briefly, CF AECs were seeded onto glass coverslips and cultured for 7 days. For virus coinfection studies, CF AECs were infected with RSV for 24 h prior to addition of bacteria. For imaging, control or RSV-infected CF AECs on coverslips were transferred to FCS2 live-cell imaging chambers (Bioptechs), stained with Hoechst stain (Molecular Probes), and then infected with S. aureus USA100 expressing GFP. Bacteria were allowed to attach for 1 h without flow and then grown for up to 6 h in MEM under flow conditions (rate of 50 ml/h). Images from 5 fields per chamber were acquired at desired time points on a Nikon Ti inverted wide-field microscope. Bacterial biomass was quantified with Nikon Elements software.

### Conditioned medium biofilm assays.

CM was collected from the apical surface of polarized CFBE41o- cells (control or preinfected with RSV) by incubating 0.5 ml MEM without phenol red supplemented with l-glutamine on the apical surface of polarized cells on transwell filters for 16 h and then collecting the liquid. A low-speed spin was performed (1,400 rpm for 10 min) to remove cell debris from CM before inoculating with bacteria. For imaging, CM was then inoculated with 7 µl of USA100-GFP (OD_600_ normalized to 0.5 in MEM). Biofilms were grown for 6 h on glass-bottom dishes (MatTek Corporation) at 37°C, 5% CO_2_. z-stack images of 5 random fields were taken for each dish on a Nikon Ti inverted microscope. Nikon Elements imaging software version 4.11 was used to measure biofilm biomass, and total biomass was calculated as the biofilm volume divided by area (expressed as cubic micrometers/square micrometer).

Microtiter plate biofilm assays were performed with control and RSV CM collected as described above. Total protein content in CM was measured with the Pierce 660-nm protein assay reagent (Thermo Scientific). Proteinase K treatment was performed by adding proteinase K (New England BioLabs; final concentration, 50 U/ml) to CM, incubating the mixture for 1 h at 37°C, and heat inactivating it at 95°C for 10 min. Filter centrifugation was performed using Amicon Ultra 3K 0.5-ml centrifugal filters (EMD Millipore). CM (0.5 ml) was applied to the filter and centrifuged at 13,000 × *g* for 10 min. Resulting medium in the filtrate and concentrate was measured and brought back up to the starting volume of 0.5 ml with MEM without phenol red supplemented with l-glutamine for subsequent biofilm assays. CM was inoculated with S. aureus (washed in MEM and diluted 1:50), and 100 µl was aliquoted per well of a 96-well flat clear-bottom plate. Biofilms were grown for 16 h with shaking at 37°C. To develop the assay, CM and planktonic bacteria were removed from wells, and wells were washed with MEM, stained for 30 min with 1% crystal violet, washed with MEM, and destained with 80:20 ethanol-acetone solution. Absorbance was measured at 550 nm to approximate biomass.

### Metal assays.

Total metal ion levels in control and RSV CMs were determined using QuantiChrome assay kits for iron, copper, and zinc (BioAssay Systems). Values shown are an average from three biological replicates, with two technical replicates performed per sample.

### TEER measurements.

Transepithelial electrical resistance (TEER) was measured in CF AECs and primary CF HBE cells on transwell filters using an EVOM2 epithelial voltohmmeter (World Precision Instruments, Inc.). Resistance measurements were recorded in milliohms and taken according to the manufacturer’s instructions.

### Dual host-pathogen RNA sequencing.

CFBE41o- cells were cultured on 75-mm transwell inserts. RNA was extracted from uninfected CFBE41o-, RSV-infected, S. aureus-infected, and RSV-*S. aureus*-coinfected static biofilm cocultures grown for 7 h by phenol-chloroform extraction using RNA-Bee (Amsbio) and 0.1-mm zirconia-silica beads in a Mini-Beadbeater (as described above for RNA extraction for quantitative PCR [qPCR]). RNA was precipitated with isopropanol and linear acrylamide, and pellets were washed with ethanol. RNA was then treated with Turbo DNase (Ambion) and purified by RNA Clean and Concentrator (Zymo). RNA concentration was measured by NanoDrop. rRNA was depleted from all samples using the Ribo-Zero Gold rRNA removal kit (Illumina), sequencing libraries were prepared using a TruSeq stranded total RNA kit, and single-end sequencing was performed with a NextSeq 500 sequencer.

Reads were processed and mapped to the human genome using CLC Genomics Workbench (v. 10.0.1; Qiagen) using standard settings, with the maximum number of hits per read set to 1. For host reads, differential expression analysis was also performed in CLC Genomics Workbench. Statistically significant changes were considered those with a *P* value of ≤0.05. For S. aureus, reads were processed and mapped to the S. aureus N315 genome in Rockhopper (v. 2.0.3), and differential expression analysis was also performed in Rockhopper. CF AEC transcriptomes were evaluated in Ingenuity Pathway Analysis software version 01-12 (Qiagen) with a license through the University of Pittsburgh Health Sciences Library System. Statistically significant changes were considered those with a *q* value of ≤0.05. Key pathways found to be differentially regulated during coinfection were mapped in Proteomaps (https://www.proteomaps.net/) (21) using fold change gene expression values.

### Data availability.

Dual host-pathogen RNA sequencing data have been deposited in the NCBI Sequence Read Archive with the BioSample accession numbers SAMN09606965, SAMN09606973, SAMN09606980, and SAMN09606993.
