# Supplementary figures and images for: Staphylococcus aureus Biofilm Growth on Cystic Fibrosis Airway Epithelial Cells Is Enhanced during Respiratory Syncytial Virus Coinfection
Source: mSphere. 2018 Aug 15;3(4):e00341-18. doi: 10.1128/mSphere.00341-18 (PMC6094059; doi:10.1128/mSphere.00341-18)

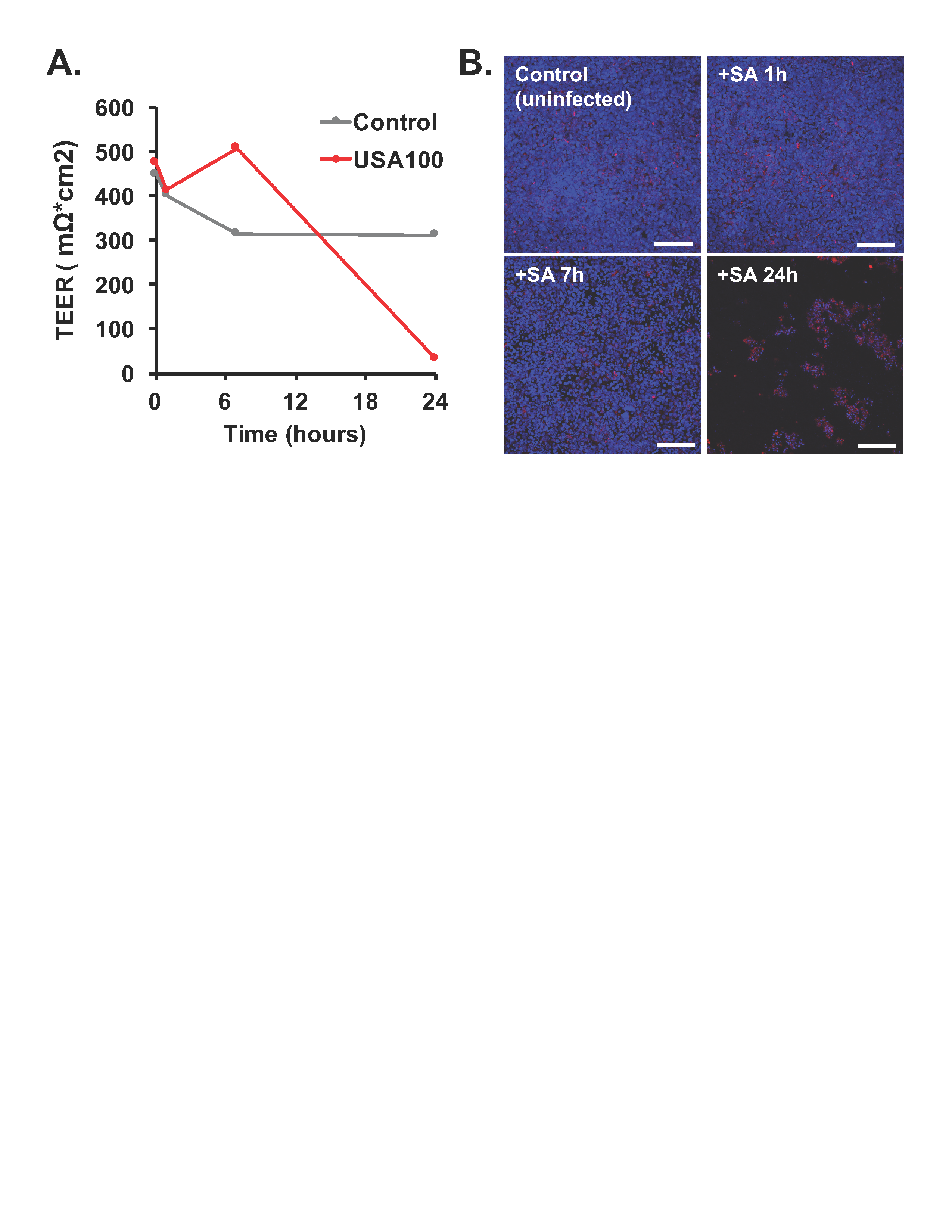

Supplement: FIG S1 [file sph004182615sf1.tif]
